# Enhancing Biological Nitrogen Fixation Through Diverse Pasture Swards

**DOI:** 10.3390/plants14172727

**Published:** 2025-09-02

**Authors:** Rukshagini Sutharsan, Paramsothy Jeyakumar, Lucy Burkitt, Dumsane Themba Matse, Ramadoss Dhanuskodi, James Hanly, Daniel J. Donaghy

**Affiliations:** 1 School of Agriculture and Environment, Massey University, Private Bag 11 222, Palmerston North 4442, New Zealand; r.sutharsan@massey.ac.nz (R.S.); l.burkitt@massey.ac.nz (L.B.); j.a.hanly@massey.ac.nz (J.H.); d.j.donaghy@massey.ac.nz (D.J.D.); 2 Crops, Environment and Land Use Programme, Teagasc, Environment, Soils and Land Use Department, Y35 TC97 Wexford, Ireland; dumsane.matse@teagasc.ie; 3 Resilient Agriculture, AgResearch, Palmerston North 4442, New Zealand; r.dhanushkodi@massey.ac.nz

**Keywords:** biological nitrogen fixation, diverse pasture, ^15^N natural abundance, nitrogen difference method, regenerative agriculture

## Abstract

Regenerative agricultural practices emphasize the use of diverse pasture species within sustainable agriculture production systems. The inclusion of a range of legume species in diverse pasture swards is likely to increase biological N fixation (BNF) across seasons, reducing the system’s reliance on synthetic N inputs. The present field study aims to quantify BNF in selected legume species within diverse pasture (combining 9 species) and standard pastures (ryegrass and clover combination) and assess their performance to identify the potential for improving N supply while maintaining year-round pasture quality. A year-round seasonal BNF was assessed by evaluating soil N status, nodulation patterns, plant composition, and conducting ^15^N natural abundance studies. The results revealed that the diverse pasture sward produced 5.4% more dry matter compared to the standard pasture, while soil mineral N (NO_3_^−^, NH_4_^+^) remained statistically similar between the two treatments. Nitrogen yield was 9.3% higher in the diverse pasture than in the standard pasture. ^15^N natural abundance analysis assessment revealed no substantial variation in BNF rates across treatments throughout the study. However, in contrast to standard pasture, the BNF rate in diverse pasture experienced a 3-fold increase from winter to summer, while the standard pasture exhibited a 1.5-fold increase. In both pasture systems, BNF increased with clover proportion up to 30%, indicating optimal fixation at moderate clover levels. The findings underscore the potential of diverse pastures when strategically managed to enhance seasonal BNF while sustaining pasture productivity.

## 1. Introduction

Grassland occupies approximately 40% of the Earth’s terrestrial land and forms an important component of the world’s food supply through livestock production. Livestock production is a vital economic activity worldwide, contributing approximately 50% of the global agricultural gross domestic product. Grass-based pastures (Ryegrass [*Lolium perenne*] and white clover [*Trifolium repens*]) are fundamental to the sustainability of livestock production, providing high-quality forage that supports efficient animal growth and productivity [1,2,3]. However, to maintain high pasture yield and quality, synthetic nitrogen (N) fertilizers are typically applied. For example, in New Zealand dairy farm systems, fertilizers are generally applied at 100–190 kg N ha^−1^ year^−1^ to sustain animal forage demand [4]. In addition, dairy animals grazing N-rich ryegrass and white clover pastures excrete 75–90% of consumed N in urine, leading to urine patches with N concentrations of 200–2000 kg N ha^−1^ [5]. However, from the applied N, less than 50% is utilized by the pasture [6]. Nitrogen surplus exceeding the pasture uptake capacity, resulting from N fertilizer application and urine patches, is lost through different avenues, such as ammonia (NH_3_) volatilization [7], nitrate (NO_3_^−^) leaching [8,9] and nitrous oxide (N_2_O) emission [10,11], often associated with environmental degradation, such as water quality deterioration, biodiversity loss [12], and greenhouse gas emissions [13].

Recently, several pasture-based management practices have been introduced to farmers to reduce N losses through fertilizer use and animal excretion [14]. Regenerative agricultural management focuses on restoring ecosystem health and vitality while increasing resilience to climate change [15,16]. Recent research highlights growing interest in regenerative management in pastoral agriculture systems, such as rotational grazing, diverse pasture swards (combining Poaceae, Fabaceae, and other herbaceous species), minimal soil disturbance, and reduced synthetic input use [17,18,19,20,21]. Implementation of diverse pasture systems under regenerative management practice holds promise for mitigating N losses and enhancing biological nitrogen fixation (BNF), strategic grazing management, and soil health enhancement strategies [20,22,23].

Multiple legume species within a diverse pasture swards can enhance total BNF, while also transferring fixed N to neighboring non-legume plants [24,25,26]. Furthermore, varied root architectures and plant traits in diverse mixtures can stimulate soil microbial communities, including free-living N-fixing bacteria [27,28], and non-leguminous forbs, such as plantain (*Plantago lanceolata*) and chicory (*Cichorium intybus*), may indirectly support BNF by improving soil conditions [29,30].

Although BNF serves as a pivotal mechanism for the natural provision of N, research on its effectiveness in diverse pasture-based regenerative systems for year-round production remains limited. Understanding how seasonal factors, such as temperature and soil moisture, affect the activity of microbes driving BNF is essential. It is hypothesized that N supplied through enhanced BNF in diverse pasture management systems could substantially offset the use of synthetic fertilizers in grazed pastures. This study aims to quantify the N contribution of legume species within a diverse pasture sward under regenerative management and investigate the seasonal variation of BNF rates by monitoring soil N concentrations and legume growth parameters over multiple seasons. Findings will provide valuable insights into optimizing pasture composition and management to enhance BNF, reduce synthetic fertilizer use, and sustain productive pasture systems.

## 2. Results

### 2.1. Dry Matter Production

**Figure 1:**
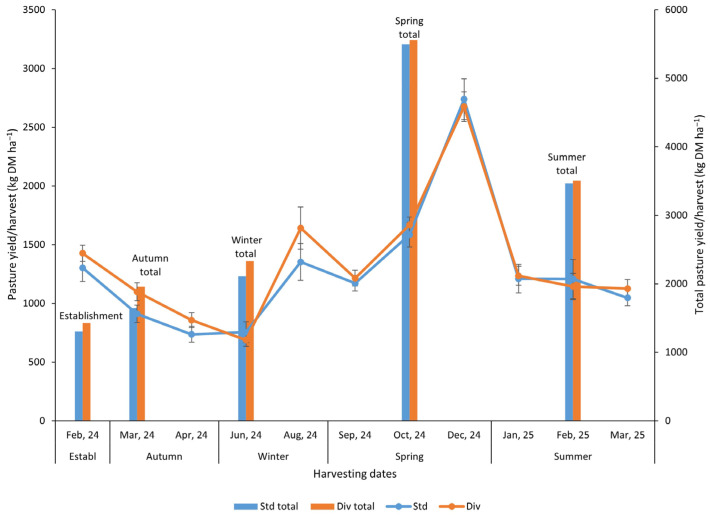
Pasture yield (kg DM ha^−1^) on every harvesting date and total pasture yield for each season (bar chart) in standard (Std) and diverse (Div) pasture treatments throughout the study. The error bar represents the standard error (n = 6). Note: Pasture yield (kg DM ha^−1^) did not show any significant differences (*p* < 0.05) between the pasture systems in each of the harvesting events.

Dry matter (DM) production in each harvesting period did not show significant differences (*p* < 0.05) between standard and diverse pastures (Figure 1). However, there were some noticeable differences between the two pasture yields at specific harvesting periods. For example, in the first harvest, the diverse pasture treatment DM yield was 9.5% higher than the standard pasture. In the second, third, and fifth harvest, diverse pasture DM yield was 21%, 16%, and 21% higher, respectively, relative to the standard pasture treatment. In terms of total DM yield in each season, the diverse pasture had a remarkably higher DM yield than the standard pasture in autumn (18.7%) and winter (11%) seasons; only a 1.2% increase was observed in both spring and summer, respectively, compared to the standard pasture sward (Figure 1).

In reference to annual cumulative yield (Figure S1), the diverse pasture treatment recorded total yields of 14.8 t DM ha^−1^ year^−1^, which represents a 5.4% increase in yields compared to standard pastures.

### 2.2. Pasture Composition

**Figure 2:**
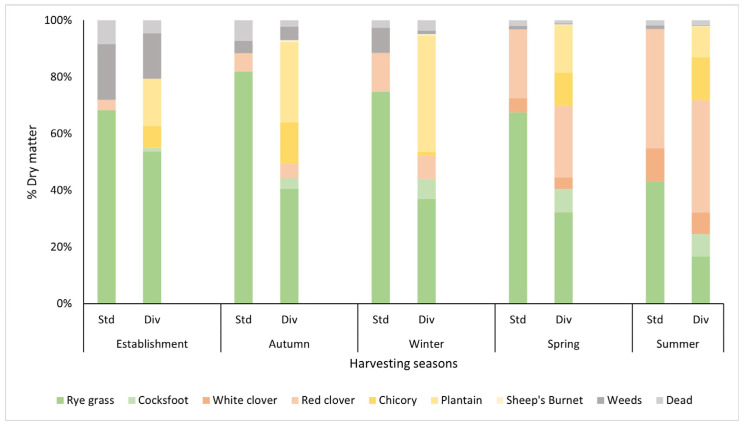
Pasture composition (%) of each species across harvest seasons in standard (Std) and diverse (Div) pasture treatments throughout the study. The X-axis represents the harvest date, while the Y-axis represents the percentage of dry matter contributed by each pasture species. Grasses include ryegrass (*Lolium perenne*) and cocksfoot (*Dactylis glomerata*); legumes include white clover (*Trifolium repens*), red clover (*Trifolium pratense*); other herbaceous species include plantain (*Plantago lanceolata*), chicory (*Cichorium intybus*), and sheep’s burnet (*Sanguisorba minor*). Weeds represent plant species not part of the sown seed mix, and dead material represents samples with over 50% dry, brown plant material.

From autumn to summer, a continuous decline in ryegrass proportion was observed in both pasture treatments. In the standard pasture treatment, the proportion of ryegrass decreased from 82% in autumn to 43% by summer, corresponding to a 50% reduction. In the diverse pasture treatment, ryegrass declined from 41% to 17% over the same period, representing a 60% reduction (Figure 2). In both pasture swards, white clover did not establish after being sown, but it started to appear in both treatments in spring from the existing seed bank in the soil. In summer, white clover contributed 11% and 8% DM to the standard and diverse swards, respectively. Under both treatments, the red clover content increased progressively throughout the seasons, with high production observed in summer, with this species contributing 42% of the DM in the standard pasture and 40% in the diverse pasture.

In diverse pastures, cocksfoot proportions remained low, ranging between 1 and 8%. It performed well in spring and summer, contributing 8% to the overall sward (Figure 2). Vetch and sainfoin did not appear throughout the study, with their absence indicating either poor germination or competition from other species. Chicory performed well in autumn and summer and made up 15% of the diverse pasture sward. It decreased in winter (1.5%) and then increased in spring (12%). Plantain performed well in autumn (28%) and winter (41%). Plantain production decreased in spring (28%) and then further declined in summer (11%). Sheep’s burnet was present at low proportions across all seasons, peaking in autumn (0.7%) and gradually declining through winter (0.5%), spring (0.2%), and summer (0.08%). Unclassified weed presence was high (18%) at establishment, but with subsequent harvests, it was reduced over time to 1.6% (Figure 2).

### 2.3. Soil Mineral N

**Figure 3:**
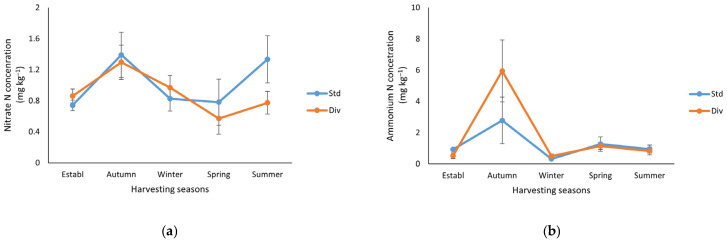
Soil mineral N (mg kg^−1^) changes at each harvesting season in standard (Std) and diverse (Div) pasture treatments throughout the study. (**a**) Nitrate concentration and (**b**) ammonia concentration. The error bar represents the standard error (n = 6). Note: Both nitrate N and ammonium N concentrations did not show any significant differences (*p* < 0.05) between the pasture systems in each season.

Soil NO_3_^−^ concentrations showed a significant decline (*p* < 0.05) from baseline levels of 12 mg kg^−1^ in both pasture systems to below 1.4 mg kg^−1^ by autumn (Figure 3a). The soil NO_3_^−^ concentrations in standard and diverse pastures were reduced to 0.8 and 0.6 mg kg^−1^, respectively, by spring. Subsequently, soil NO_3_^−^ levels exhibited a seasonal increase during the summer months. Even though these variations were not statistically significant (*p* < 0.05), the NO_3_^−^ concentration in the standard pasture was 60% higher compared to the diverse pasture.

Soil NH_4_^+^ concentrations decreased from the baseline measurement (4 mg kg^−1^) and subsequently increased to a sharp peak in autumn. This peak reached 2.8 and 6.0 mg NH_4_^+^ kg^−1^ in the standard and diverse pasture, respectively. The NH_4_^+^ concentration sharply decreased to approximately 0.5 mg NH_4_^+^ kg^−1^ in both treatments during winter, with a slight increase observed in spring (1.3 mg NH_4_^+^ kg^−1^) and summer (1 mg NH_4_^+^ kg^−1^) in both treatments (Figure 3b).

### 2.4. Pasture N Yield

The cumulative N yield across all species and seasons was higher in the diverse pasture (277.5 kg N ha^−1^) compared to the standard pasture (258 kg N ha^−1^). Ryegrass was the dominant contributor to N yield in the standard pasture sward, ranging from 20.6 to 44.8 kg N ha^−1^, with the highest value observed in spring. The cumulative N yield by ryegrass in the standard pasture was approximately 2-fold, with 147.1 kg N ha^−1^, in contrast to 79.5 kg N ha^−1^ in the diverse pasture. This reduction in ryegrass uptake in the diverse sward can be attributed to the presence of other species.

In both pasture systems, cumulative N yield by red clover was comparable, with totals of approximately 85 kg N ha^−1^. However, the highest uptake contribution was observed in both standard and diverse pasture during spring and summer, each approximately 40 kg N ha^−1^. This observation aligns with the seasonal variations in pasture production across the treatments. White clover N yield was highest in the spring and summer seasons. Summer uptake was 2-fold higher in the standard pasture (16.03 kg N ha^−1^) compared to the diverse pasture (8.17 kg N ha^−1^) (Table 1).

Cocksfoot N yield in the diverse sward, increasing from 1.8 kg N ha^−1^ in autumn to 5.7 kg N ha^−1^ in summer, with a cumulative uptake of 14.2 kg N ha^−1^ (Table 1). Chicory uptake was lowest in winter (0.6 kg N ha^−1^) and highest in both spring and summer at 14.1 kg N ha^−1^, with a cumulative N yield of 39.7 kg N ha^−1^, highlighting its significant role in warmer seasons. Plantain N yield was consistently stable, ranging from 7.9 to 13.8 throughout the seasons, contributing to a total of 44.3 kg N ha^−1^ (Table 1).

### 2.5. Biological N Fixation Measurements by Nodule Number and ^15^N Natural Abundance Techniques

Biological N fixation was quantified using three distinct methodologies: nodule count, N difference calculation, and ^15^N natural abundance analysis. The hypothesis was formulated that all three methods would yield comparable patterns across treatments and seasons. While the N difference and ^15^N natural abundance methods exhibited consistent patterns across treatments and seasons (*p* < 0.05; r = 0.838) (Figure S2), the nodule count method deviated from this alignment.

Nodule counts in both standard and diverse pastures decreased in winter and summer, with a significant increase (*p* < 0.05) in spring (Table 2). Despite the insignificant difference, the diverse pasture consistently had higher nodule counts throughout the seasons.

The N difference and ^15^N natural abundance methods estimate BNF in the same unit, but the recovery rate is much lower in the N difference method. Unlike the N difference method, which compares total plant N between fixing and non-fixing species, the isotope method directly traces the proportion of N derived from atmospheric N_2_. Overall, BNF varied significantly (*p* < 0.05) with seasons but showed no significant difference (*p* < 0.05) between standard and diverse pastures within a season. The BNF was lowest (1.6 to 1.8 kg N ha^−1^) in the autumn season. In winter, BNF increased fourfold in the standard pasture system and doubled in the diverse pasture system; however, only the increase in the standard pasture was statistically significant (*p* < 0.05). Although there was no significant difference (*p* < 0.05) between treatments, the standard pasture fixed twice as much N as the diverse pasture during this period. Diverse pasture showed a threefold increase (11.3 kg N ha^−1^), while the standard pasture recorded an 87% increase (13.1 kg N ha^−1^) in spring compared to winter. In standard pasture, fixation was reduced by 18% in summer; however, the diverse pasture did not show a reduction in summer (Table 2).

## 3. Discussion

### 3.1. Dry Matter Production and Pasture Composition

The DM production observed in the present study indicates a seasonal pattern in both standard and diverse pastures, with fluctuations likely influenced by environmental conditions, such as temperature, rainfall, and soil moisture availability. The initial decline in DM production from autumn to winter aligns with cooler temperatures and potentially reduced plant growth rates, followed by a recovery in early spring and a peak in late spring, likely driven by favorable growing conditions. While DM production per harvest was not always significantly different between standard and diverse pastures, the diverse mixtures frequently outperformed the standard sward in specific seasons, particularly in autumn and winter, with 18.7% and 11% higher DM yields, respectively. This resulted in a 5.4% greater annual cumulative yield for the diverse pasture (14.8 t DM ha^−1^ year^−1^). These findings align with research showing that, although cumulative DM yield differences between standard and diverse pastures may not always be statistically significant, diverse swards can outperform standard ryegrass–clover pastures in certain seasons or years, particularly when functional groups such as plantain and legumes are abundant [31,32]. For example, Graham et al. [32] reported that diverse pastures could yield up to 27% more than ryegrass–clover in some years, corresponding with increased proportions of plantain and clover.

The pasture botanical composition results suggest that species diversity alone did not enhance productivity under the no-N conditions applied in this study, with DM yield largely influenced by seasonal climatic variations. Ryegrass primarily supported DM production in the standard pasture, while a combination of grasses and other herbaceous species contributing to productivity in the diverse pasture, with plantain showing higher production in winter and chicory peaking from late spring to early summer, consistent with previous studies highlighting their role in sustaining summer and winter productivity [31,33]. While diverse pastures can enhance productivity, the magnitude of this effect depends on factors, such as species interactions, nutrient availability, and seasonal dynamics [2,34]. White clover, though slow to establish in autumn and winter, re-emerged from the seed bank and contributed modestly to summer yields. The increment of white clover in the pasture botanical composition is positively related to the temperatures and soil water availability, being minimal in winter (<10%) and increasing towards the summer until a point when the soil water availability becomes a limiting factor for its growth [35]. Vetch and sainfoin failed to establish, likely due to poor germination or competitive suppression, underscoring the importance of species selection and establishment practices [36].

### 3.2. Soil Mineral N in Pasture Systems

In the present study, no N fertilizer was applied during the pasture production. The pronounced initial decline in soil mineral N levels from baseline to first pasture cut (Table 3 and Figure 3) suggests rapid plant uptake or possible leaching losses following the start of pasture growth. The consistently low N levels throughout the study indicate that N mineralization rates were likely low or that NO_3_^−^ was efficiently taken up by plants as soon as it became available [37]. These low N levels align with the period of peak plant growth and N demand, as observed in similar studies [38].

During summer, soil NO_3_^−^ concentrations increased in both systems, likely due to reduced plant uptake, as growth slowed and soil mineralization rates increased with warmer temperatures. Although these seasonal fluctuations were not statistically significant, the standard pasture plots maintained NO_3_^−^ concentrations approximately 60% higher than those in the diverse pasture. This trend suggests that diverse pastures may be more effective at taking up soil NO_3_^−^, possibly due to complementary rooting patterns, which can enhance N uptake and cycling [38,39].

The pronounced NH_4_^+^ peak observed in autumn in the diverse pasture suggests that the extensive root system and favorable environmental conditions for microbial activity may result in elevated OM decomposition rates. The subsequent decline in NH_4_^+^ levels during winter suggests a concomitant process of NO_3_^−^ conversion and plant uptake, coinciding with a decrease in microbial activity. Lower NH_4_^+^ levels during the spring and summer are likely attributed to the enhanced plant N uptake and DM production. However, there are no significant differences between treatments, indicating that both pasture systems followed similar N cycling dynamics under the given conditions. Additionally, our present measurements of soil mineral N may not fully represent the mineral N, as organically bound N forms have also been shown to contribute to N uptake in pasture [40].

### 3.3. Seasonal Changes in Pasture N Yield

The results indicate that the diverse pasture exhibited a marginally elevated cumulative N yield rate (277.5 kg N ha^−1^) compared to the standard pasture (258.0 kg N ha^−1^). This result reinforces the productivity advantage of diverse swards observed in DM yield, particularly during autumn and winter, and highlights the role of species complementarity in enhancing total N capture from the soil [38,41]. A twofold reduction in N yield by ryegrass in the diverse pasture in comparison to standard pasture ryegrass N yield reflects the competitive presence and contribution of other functional groups, a finding consistent with Sanderson et al. [39]. The reduction in ryegrass N yield in the diverse sward is compensated by competitive N yield from species such as chicory, plantain, and cocksfoot, which show different ecological niches and seasonal growth patterns [38,41]. For example, chicory’s N yield was highest in summer, while plantain contributed most to autumn and winter N yield, supporting the concept of temporal variation among species [41].

### 3.4. Biological N Fixation

The differences in BNF between the two pasture management systems were not statistically significant throughout the experiment. While nodule number indicated a higher N fixation rate in diverse pasture swards compared to the standard, this was not reflected in the other two methods. Nodule number is a point in time measure and does not reflect the actual N fixed over time, as not all pink nodules are viable or efficient. In contrast, ^15^N natural abundance and N difference methods provide integrated, quantitative estimates of BNF over time [42]. The N difference method likely underestimates BNF because legumes often take up less soil N than the reference plant, and analyses restricted to shoot N omit N stored in roots and nodules [43,44]. In our study, although both the N difference and ^15^N isotope methods measured only shoot N, BNF estimates from the N difference method were consistently lower than those from the isotope method, reflecting the method’s inherent underestimation. Consequently, the total fixed N in the plant–soil system may be higher than reported here. Winter BNF was higher in both pasture systems than in autumn, as measured by ^15^N natural abundance and N difference methods. The lower autumn BNF may have been caused by intense summer (previous) weed competition during pasture establishment, which reduced the initial clover composition. Furthermore, during pasture establishment, root development and microbial activity may have increased soil mineralized N in autumn, making clover rely more on soil N uptake than BNF. However, by winter, increased plant uptake may decline soil mineralized N, forcing legumes to depend more on BNF. In spring and summer, when N yield was highest (around 90 kg N ha^−1^), BNF activity also peaked, as shown by elevated ^15^N natural abundance values (11–13 kg N ha^−1^) in both pasture systems. This seasonal pattern aligns with previous findings by Ledgard et al. [45], who reported variations in N fixation rates in New Zealand pastures throughout the year. The BNF activity is much lower in winter than spring and summer, due to reduced plant metabolism and rhizobium activity from cold temperatures [46,47], limited photosynthesis for energy supply [48], and waterlogged soils that hinder nodule function [49].

Despite the expectation that diverse pastures would support higher BNF due to increased plant diversity [50], the standard pasture showed higher N fixation in winter. The high clover composition (68% higher) in the standard pasture compared to the diverse pasture sward likely contributed to this higher N fixation. Obviously, other species, particularly plantain (40%), competed for resources, reducing clover establishment and fixation in the diverse pasture. This finding aligns with previous research suggesting that the presence and productivity of legumes, rather than overall plant diversity, are the primary drivers of BNF in temperate pastures [43,45]. The diverse pasture exhibited a steep threefold increase in BNF during spring, which was sustained through summer. In contrast, the standard pasture showed a more modest increase, with 87% in spring and 53% in summer compared to winter values. The greater increase in BNF in the diverse pasture is supported by the equal clover composition observed in both pasture systems during spring and summer, in contrast to a 68% lower clover presence in the diverse pasture during winter [51]. Although our initial hypothesis posited that deep-rooted species would deplete soil N and thereby stimulate BNF by red clover, this effect was not observed within the timeframe of this study. This may be due to insufficient establishment of these species or the short duration since establishment. Similar N yield and soil N availability between the two pasture types further explain the lack of significant differences in BNF. It is important to note that no synthetic fertilizers were applied during the study, in line with regenerative management principles. While this approach supports long-term soil health and sustainability, it may have limited overall pasture productivity and masked potential differences in N cycling between systems [52]. Additionally, the absence of fertilizer N inputs likely contributed to the non-significant differences in dry matter yield, N yield, and BNF between the standard and diverse pastures.

**Figure 4:**
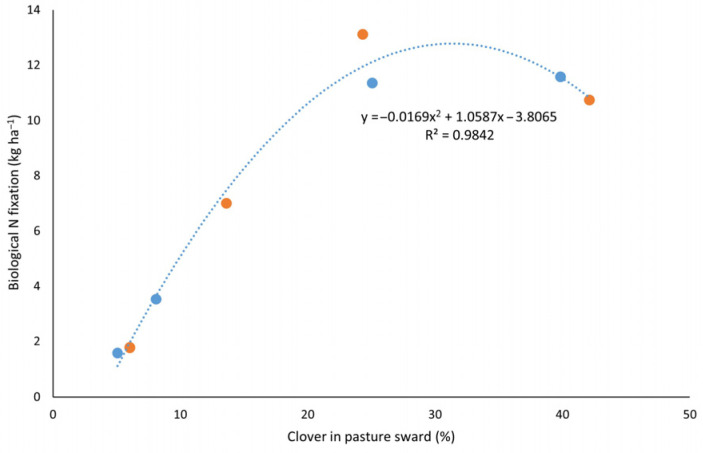
The relationship between clover fraction in pasture sward and the BNF in combined standard (●) and diverse (●) pasture systems.

The relationship between clover proportion and BNF followed a non-linear trend in both standard and diverse pastures (Figure 4). As clover composition increased, BNF also rose, reaching an apparent optimum at around 30% clover cover. This suggests that moderate clover presence effectively supports symbiotic N fixation, likely due to optimal resource competition and balanced species interactions. However, when clover cover exceeded 35%, BNF plateaued and began to decline. This reduction may be attributed to increased N transfer from legumes to the soil, which, in the context of a reduced non-legume population, could not be effectively utilized [25,53]. As a result, mineral N accumulation in the soil may have suppressed further N fixation by legumes through feedback inhibition. These findings underscore the importance of maintaining a balanced clover composition in mixed sward systems to optimize N fixation and nutrient use efficiency.

## 4. Materials and Methods

A plot study was designed to evaluate the N contribution of individual legume species within a diverse pasture sward to observe seasonal variation in BNF rates by monitoring soil and plant N concentrations and legume growth parameters, including nodule number and dry matter, over multiple seasons in comparison to the BNF in standard pasture.

### 4.1. Experimental Site and Soil Characterization

**Figure 5:**
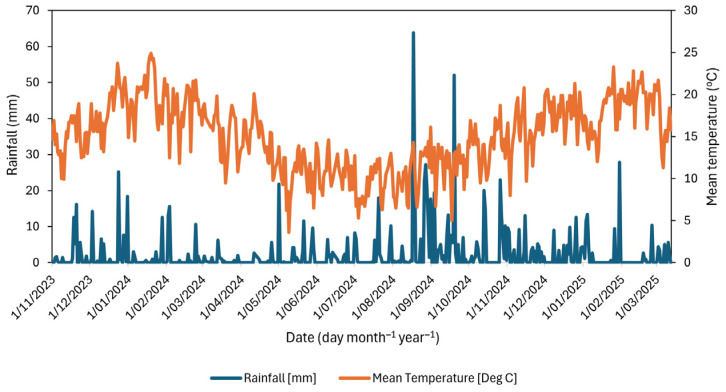
Daily total rainfall (mm) and mean air temperature (°C) at the study site during the experimental period from 1 November 2023 to 12 March 2025. Climatic data for the experimental period at the site was downloaded from the National Institute of Water and Atmosphere (NIWA) database [54]. The NIWA data for the Manawatū site was sourced from station number 3925, which is located approximately 300 m from the experimental site.

This field study was conducted at Massey University Pasture and Crop Research Unit, located on Poultry Farm Road, 5 km south of Palmerston North, NZ (40.38177° S, 175.60813° E), which has a temperate climate. The average daily temperatures in Manawatū range from 20 to 22 °C in summer to approximately 12 °C in winter, with an annual average rainfall of 984 mm over 30 years [54]. The average daily rainfall at the study site was 1.1, 10.3, 3.5, and 1.7 mm day^−1^ in autumn, winter, spring, and summer, respectively. The corresponding seasonal accumulated rainfall totals were 93.4, 917.3, 321.2, and 148.8 mm. Average temperatures for the respective seasons were 13.3 °C, 3.4 °C, 13.7 °C, and 18.4 °C. The highest daily rainfall was recorded in winter on 17 August 2024 (63.8 mm), followed by spring on 19 September 2024 (52.0 mm) (Figure 5).

The predominant soil type at the study site is the Manawatū Recent soil, according to the New Zealand soil orders, and Dystric Fluventic Eutrudept, according to the U.S. Soil Taxonomy classification. The soil texture is silt loam, and it is well-drained [55]. Before sowing, baseline soil samples were collected at a depth of 7.5 cm to determine basic soil characteristics (Table 3). In each plot, 10 soil cores (7.5 cm depth × 2 cm diameter) were taken along a zigzag transect and bulked for basic soil analysis. Soil moisture was determined gravimetrically by oven-drying samples at 105 °C for 48 h. Soil pH was measured using air-dried soil in a 1:2.5 soil-to-water suspension with a pH meter. Extractable aluminum (Al) and iron (Fe) were determined via acid ammonium oxalate extraction [56] and analyzed using microwave plasma atomic emission spectroscopy (4200 MP-AES, Agilent, Santa Clara, CA, USA). Soil NO3- and ammonium (NH_4_^+^) were extracted using 2 M potassium chloride (KCl) and analyzed using an autoanalyzer [56]. Olsen P was extracted with 0.5 M sodium hydrogen carbonate (NaHCO_3_) and measured using the ammonium molybdate colorimetry method. Exchangeable cations (Ca, Mg, Na, and K) were extracted using the semi-micro leaching method and analyzed via MP-AES (Agilent, USA) to determine CEC. Total N and carbon (C) concentrations were analyzed using a Vario MACRO Cube CHNS elemental analyzer (Elementa Anlysensysteme GmbH, Hanau, Germany). The soil had a pH of 5.93, a cation exchange capacity (CEC) of 15.64 cmol_(+)_ kg^−1^, NO_3_^−^ concentration of 11.82 mg kg^−1^, and Olsen phosphorus (P) concentration of 46 mg kg^−1^ (Table 3). More detailed results are shown in Table 3.

### 4.2. Field Preparation and Experimental Design

This experiment was conducted from spring 2023 to late summer 2025 (1 November 2023 to 12 March 2025). In preparation for this study, the site was sprayed with Weed master 360 (360 g L^−1^ of glyphosate [N-(phosphonomethyl)glycine]) at a rate of 3 L ha^−1^, followed by ploughing and secondary cultivation. No basal fertilizer was applied to the plots before planting on the 1st of November 2023, and no fertilizer was added throughout the study period. In this study, two pasture treatments, standard and diverse pastures, were evaluated. The standard pasture seed mix was comprised of white clover, red clover, and perennial ryegrass (Poaceae and Fabaceae family). The diverse pasture swards were comprised of grasses, legumes, and other herbaceous species (Poaceae, Fabaceae, Plantaginaceae, Asteraceae, and Rosaceae families). More details about each pasture species sowing rates and each species cultivar are detailed in Table 4.

**Figure 6:**
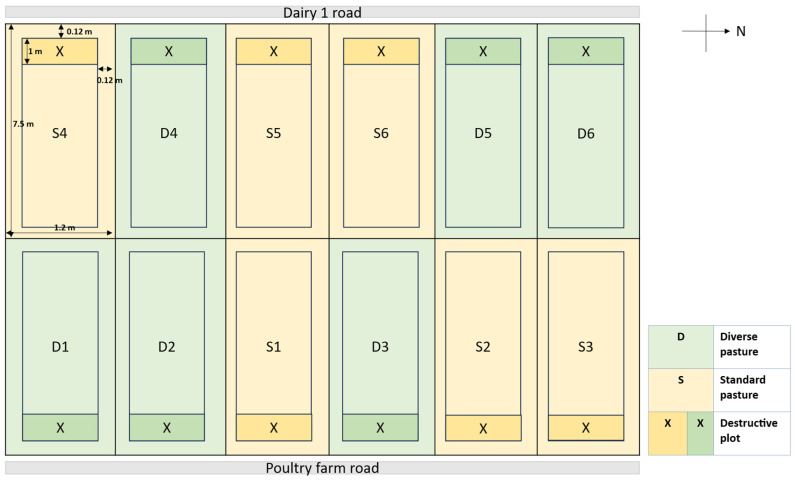
Experimental layout includes two treatments: standard pastures (pasture swards comprised of white clover (*Trifolium repens*), red clover (*Trifolium pratense*), and perennial ryegrass (*Lolium perenne*)) and diverse pastures (pasture swards comprised of grasses (ryegrass (*Lolium perenne*) and Cocksfoot (*Dactylis glomerata*)), legumes (white clover (*Trifolium repens*), red clover (*Trifolium pratense*), vetch (*Vicia sativa*), sainfoin (*Onobrychis viciifolia*)), and other herbaceous species (plantain (*Plantago lanceolata*), chicory (*Cichorium intybus*), sheep’s burnet (*Sanguisorba minor*)). Each with 6 replicates arranged in a randomized block complete design. Not to scale.

The pastures were established in plot sizes measuring 7.5 m × 1.2 m (9 m^2^). The treatments were arranged in a randomized complete block design (RCBD), and each treatment was replicated six times. A narrow strip within each paddock was designated (1.2 m^2^) for destructive pasture sampling. Between each paddock and the edge of the paddock, there was 0.12 m of walking space (Figure 6).

Pasture species were seeded on 1 November 2023, using an Oyjord plot drill, followed by rolling of the entire area. Seeding was carried out at rates recommended for regenerative farming, as determined by an experienced agronomist and shown in Table 4. The pasture was allowed to establish for 3 months. After establishment, all pastures were mowed to a residual height of 50 mm above the soil surface (Week 0) using a flex-wing rotary mower. This was done to allow light penetration and tillering, to ensure uniform height before the collection of data, and to minimize competition from neighboring plots.

### 4.3. Sampling and Measurements

#### 4.3.1. Pasture Sampling and Analysis

Pasture harvesting was conducted from February 2024 to March 2025, totaling eleven cuts throughout the experimental period (Table 5). Harvesting intervals were planned based on pasture growth and the physiological leaf stage of ryegrass [57]. Herbage mass (kg DM) was measured by mowing entire individual plots to a height of 50 mm using a rotary mower equipped with a rear catcher. The collected material was weighed immediately after cutting. Representative pasture subsamples were then taken, freshly weighed, and dried at 60 °C for 48 h to determine dry matter (DM) yield per hectare.

Botanical composition analysis was assessed in each plot before each harvesting period. A sub-sample of approximately 50 g fresh weight was collected from each replicate plot by cutting three strips (300 mm × 77 mm) in each plot to a standard height of 50 mm to determine the percentage of each species present at each harvest. Pasture samples were sorted manually into each species, weed (plant species that were not part of the sown seed mix), and dead material (samples with over 50% dead plant material, which were dry and brown), and their dry weights were determined after oven drying at 60 °C for 48 h. Nitrogen analysis was conducted for each species within their botanical groups, and a total sward N concentration was calculated. N yield was calculated by multiplying the pasture N concentration (%) by the pasture dry matter yield (kg DM ha^−1^).

#### 4.3.2. Soil Mineral N Analysis

Soil samples were collected and analyzed for soil mineral N during each harvesting interval (Table 5). In each plot, 10 soil cores (7.5 cm depth × 2 cm diameter) were taken along a zigzag transect and bulked for analysis. Soil mineral NH_4_^+^-N and NO_3_^−^-N concentrations were measured by extracting 5 g field-moist soil with 30 mL of 2 M KCl in an end-over-end shaker for 1 h. The resulting extraction was centrifuged at 1100× *g* for 10 min, then filtered through a Whatman 42 filter and analyzed for soil mineral NH_4_^+^-N and NO_3_^−^-N concentrations using a Seal autoanalyzer [56].

### 4.4. Biological N Fixation Measurements

#### 4.4.1. Nodule Number

For the nodule number, plants were uprooted from the destructive plots during autumn, winter, spring, and summer. Soil coring, alongside the plants, gathered the entire root systems and aboveground biomass. Loose soil was removed from the roots and washed carefully. Active pink nodules were counted from the entire root system. After that, the roots were dried at 60 °C for 48 h to obtain the root dry weight. From that nodule number, the plant root dry weight^−1^ was calculated.

#### 4.4.2. ^15^N Natural Abundance Method

After botanical composition measurement, the dried ryegrass and legumes were ground in a ball mill to a fine powder and analyzed for total N and ^15^N using a mass spectrometer.

The calculation of pNdfa for the natural ^15^N abundance samples was determined as described by Unkovich et al. [43]. The δ^15^N value was calculated as the deviation in the ^15^N concentration from atmospheric N_2_ (δ^15^N = 0.366 atom%) using Equation (1).

(1) δ15N(‰)=sample atom %N15−0.3663 0.3663 ×1000

The proportion of clover N derived from the atmosphere (pNdfa) was then calculated using ryegrass in the mixture as a reference plant, according to Equation (2):

(2) $$pNdfa(\%)=\frac{\delta^{15}N\;grass\left(‰\right)-\delta^{15}N\;clover(‰)}{\delta^{15}N\;grass\left(‰\right)-B(‰)}\times 100$$

where ‘B’ is the δ^15^N of shoots of legumes that are fully dependent upon N_2_ fixation and sampled at the same growth stage as the field plants. In this study, the B value was estimated as an apparent B value (Bapp) for the experimental site based on the lowest δ^15^N values of clover [58].

The amount of N_2_ fixed was then calculated as Equation (3):

(3) $$N_{2}Fixation\left(kg\;{ha}^{-1}\right)=\frac{\left(clover\;dry\;matter\left(kg\;{ha}^{-1}\right)\times clover\;N\;concentration\left(\%\right)\times pNdfa\left(\%\right)\right)}{10,000}$$

#### 4.4.3. N Difference Method

The amount of N_2_ fixed is calculated as the difference in N yield of the N_2_-fixing and reference plants (Equation (4)). The assumption here is that the non N_2_-fixing reference and N_2_-fixing plants extract the same amount of N from the soil [43].

(4) $$N_{2}\;fixed=N\;yield\;N_{2}\;fixing\;plant-N\;yield\;reference\;plant$$

### 4.5. Statistical Analysis

Data normality test and statistical analyses were undertaken using R (version 4.3.2). The treatment comparison effects were analyzed using ANOVA, and significant (*p* < 0.05) differences between means were determined using Duncan’s Multiple Range Test (DMRT).

## 5. Conclusions

The study demonstrated clear seasonal patterns in DM production and N dynamics across standard and diverse pasture systems, with diverse swards showing a trend of higher autumn and winter pasture productivity. While species diversity alone did not consistently enhance overall DM production under no-N conditions, the complementary growth and N yield by functional groups such as other herbaceous species and legumes contributed to improved resource capture and seasonal resilience in diverse pastures. Biological N fixation was statistically similar between pasture systems. However, fixation rates in diverse pastures significantly exceeded those in standard pastures from winter to summer, indicating that legume abundance rather than diversity primarily drives fixation rates. These findings affirm that seasonal and environmental factors, including temperature and soil moisture, strongly influenced N fixation and uptake patterns.

Environmental implications: Importantly, the study was conducted without synthetic N fertilizer inputs aligned with regenerative management goals, demonstrating the potential of well-managed diverse pastures to support productivity while reducing external N inputs. This has clear environmental implications, as enhanced BNF in place of synthetic fertilizers can contribute to more sustainable and climate-resilient pasture systems.

Overall, this research supports the value of functionally diverse swards in regenerative systems, offering a pathway to reduce reliance on synthetic N inputs through the strategic use of BNF.

Limitations of this study: It is imperative to acknowledge several limitations that should not be overlooked. The expected benefits from legume diversity in the diverse pasture sward were not fully realized, likely due to limitations in initial seed germination. The study was conducted over a single growing cycle; future research should include multiple seasonal cycles to better capture diverse pasture effects on BNF. Conducting a study under realistic grazing conditions would enhance its practical relevance and introduce variability that could potentially influence the outcomes.

Recommendations: Future studies should refine initial legume establishment strategies, expand monitoring over successive years, and enhance BNF measurement techniques to incorporate the contributions of free-living microorganisms. We also recommend establishing this research by including measurements of nitrate leachate and gaseous losses under field conditions to assess nitrogen usage efficiency of diverse pastures under regenerative management.

## Figures and Tables

**Table 1:** N yield (kg N ha^−1^) by each species across harvesting seasons in standard (Std) and diverse (Div) pasture treatments throughout the study. The table presents mean values ± standard error (n = 6). N yield was calculated by multiplying pasture N concentration (%) with pasture dry matter yield (kg DM ha^−1^).

Species	Establishment		Autumn		Winter		Spring		Summer		Cumulative N Yield	
	Std	Div	Std	Div	Std	Div	Std	Div	Std	Div	Std	Div
Ryegrass	20.6 ± 2.4 ^B^_a_	17.8 ± 1.5 ^A^_a_	29.11 ± 2.6 ^B^_a_	19.7 ± 2.8 ^A^_b_	24.2 ± 2.9 ^B^_a_	15.9 ± 3.2 ^A^_a_	44.8 ± 4.4 ^A^_a_	16.7 ± 1.6 ^A^_b_	28.3 ± 1.3 ^B^_a_	9.4 ± 1.1 ^A^_b_	147.1	79.5
Cocksfoot		*		1.8 ± 0.9 ^A^		3.1 ± 0.5 ^A^		3.6 ± 0.8 ^A^		5.7 ± 1.2 ^A^		14.2
Red clover	1.3 ± 0.7 ^C^	*	2.9 ± 1.4 ^C^_a_	2.9 ± 0.9 ^B^_a_	6.7 ± 2.3 ^C^_a_	4.2 ± 0.5 ^B^_a_	34.7 ± 4.4 ^B^_a_	40.6 ± 4.0 ^A^_a_	39.9 ± 5.4 ^A^_a_	36.4 ± 2.2 ^A^_a_	85.4	84.1
White clover	*	*	*	*	*	*	9.47 ± 1.2 ^A^_a_	7.1 ± 1.0 ^A^_a_	16.03 ± 1.5 ^A^_a_	8.17 ± 0.9 ^A^_a_	25.5	15.3
Chicory		2.3 ± 1.2 ^B^		8.6 ± 4.0 ^A^		0.6 ± 0.2 ^B^		14.1 ± 0.4 ^A^		14.1 ± 0.8 ^A^		39.7
Plantain		4.8 ± 0.4 ^C^		12.2 ± 1.8 ^A^		13.8 ± 1.5 ^A^		10.5 ± 1.1 ^AB^		7.9 ± 1.6 ^B^		44.4
Sheep burnet		*		0.3 ± 0.07 ^A^		0.2 ± 0.06 ^A^		0.1 ± 0.02 ^A^		*		0.6
Total	21.9 ± 2.5 ^C^_a_	20.1 ± 2.3 ^C^_a_	32.0 ± 3.1 ^B^_a_	45.4 ± 3.1 ^B^_a_	30.8 ± 4.2 ^B^_a_	37.7 ± 3.7 ^B^_a_	89.0 ± 8.9 ^A^_a_	92.6 ± 4.3 ^A^_a_	84.2 ± 6.3 ^A^_a_	81.6 ± 2.5 ^A^_a_	258.04	277.53

Note: Different lowercase letters in the same row indicate significant differences between treatments. Different uppercase letters in the same row indicate significant differences between seasons (*p* < 0.05). * Pasture species did not germinate/establish.

**Table 2:** Seasonal variation in biological N fixation (BNF) throughout the study period across standard and diverse pastures. Treatments include Std = standard pasture and Div = diverse pasture.

Season	Treatment	Nodule Number Root Dry Weight^−1^	N Difference (kg N ha^−1^)	^15^N Natural Abundance (kg N ha^−1^)
Autumn	Std	33 ± 5 _a_^B^	0.8 ± 0.4 _a_^C^	1.8 ± 0.8 _a_^C^
	Div	41 ± 7 _a_^B^	0.6 ± 0.3 _a_^C^	1.6 ± 0.7 _a_^B^
Winter	Std	26 ± 6 _a_^C^	3.0 ± 0.6 _a_^B^	7 ± 1.7 _a_^B^
	Div	25 ± 4 _a_^C^	1.5 ± 0.4 _a_^C^	3.5 ± 1 _a_^B^
Spring	Std	108 ± 50 _a_^A^	7.8 ± 0.9 _a_^A^	13.1 ± 0.6 _a_^A^
	Div	166 ± 32 _a_^A^	7.3 ± 0.8 _a_^A^	11.3 ± 2.1 _a_^A^
Summer	Std	37 ± 18 _a_^B^	3.1 ± 0.4 _a_^B^	10.7 ± 1.6 _a_^AB^
	Div	58 ± 11 _a_^B^	3.1 ± 0.6 _a_^B^	11.6 ± 0.7 _a_^A^

Note: Values mean ± standard error of the mean (n = 3). Different lowercase letters in the same column indicate significant differences between treatments. Different uppercase letters in the same column indicate significant differences between seasons (*p* < 0.05). The N difference was calculated as $N_{2}\;fixed=N\;yield\;N_{2}fixing\;plant-N\;yield\;reference\;plant.$

**Table 3:** Baseline chemical properties of Manawatū Recent soil analyzed before pasture establishment. Soil sampling at a depth of 7.5 cm.

Soil Parameters	Values
pH (H_2_O)	5.93 ± 0.05
Fe (mg kg^−1^)	3018 ± 256
Mn (mg kg^−1^)	81.21 ± 6.3
Al (mg kg^−1^)	773.1 ± 76
Total N (g kg^−1^)	2.1 ± 0.1
Total Organic C (g kg^−1^)	16.5 ± 01.2
Olsen P (mg kg^−1^)	45.89 ± 4.47
CEC (cmol_(+)_ kg^−1^)	15.64 ± 0.52
NO_3_^−^ (mg kg^−1^)	11.82 ±0.55
NH_4_^+^ (mg kg^−1^)	3.62 ± 0.78

**Table 4:** Pasture species and their sowing rate used in the experiment.

Sward Type	Pasture Classification	Scientific Names	Cultivar	Species (Common Names)	Seeding Rate (kg ha^−1^)
Diverse	Grasses (Poaceae)	*Lolium perenne*	Platform AR37	Diploid perennial ryegrass	9
		*Lolium perenne*	4 Front	Tetraploid hybrid ryegrass	13
		*Dactylis glomerata*	Safin	Cocksfoot	3
	Legumes (Fabaceae)	*Trifolium repens*	Kotuku	White clover large-leaved	2
		*Trifolium repens*	Weka	White clover medium/large leaves	2
		*Trifolium pratense*	Relish	Red clover	4
		*Vicia sativa*		Vetch	2
		*Onobrychis viciifolia*		Sainfoin	1
	Other herbaceous species (Asteraceae)	*Cichorium intybus*	Choice	Chicory	1
	(Plantaginaceae)	*Plantago lanceolata*	Ecotain	Plantain	2
	(Rosaceae)	*Sanguisorba minor*		Sheep’s Burnet	2
Standard	Grasses (Poaceae)	*Lolium perenne*	Platform AR37	Diploid perennial ryegrass	10
		*Lolium perenne*	4 Front	Tetraploid hybrid ryegrass	15
	Legumes (Fabaceae)	*Trifolium repens*	Kotuku	White clover large-leaved	2
		*Trifolium repens*	Weka	White clover medium/large leaves	2
		*Trifolium pratense*	Relish	Red clover	4

**Table 5:** Harvest events of standard and diverse pasture plots during the experimental period.

Growing Years	Harvesting Dates
Year 1 (2024)	
Summer	20 February during establishment
Autumn	19 March
Autumn	23 April
Winter	21 June
Winter	29 August
Spring	30 September
Spring	31 October
Spring	9 December
Year 2 (2025)	
Summer	9 January
Summer	11 February
Summer	12 March

## Data Availability

The data analyzed during this current study are not publicly available because it is part of the first author’s graduation thesis but can be made available from the corresponding author upon reasonable request.

## Supplementary Materials

The following supporting information can be downloaded at: https://www.mdpi.com/article/10.3390/plants14172727/s1, Figure S1: Cumulative dry matter production (kg DM ha^−1^) of standard and diverse pasture treatments throughout the study. The error bar represents the standard error (n = 6). Figure S2: Correlation between biological nitrogen fixation (BNF) calculated using the isotope method and the nitrogen difference (ND) method. The scatter plot includes a fitted linear regression line (y = 0.4872x − 0.2901) with an R^2^ value of 0.7042, indicating a moderate positive correlation between the two methods.
