# Supplementary material for: Enhancing Biological Nitrogen Fixation Through Diverse Pasture Swards
Source: Plants (Basel). 2025 Sep 2;14(17):2727. doi: 10.3390/plants14172727 (PMC12430658; doi:10.3390/plants14172727)
Supplement: Supplementary file 1 [file plants-14-02727-s001.zip › plants-3818132-supplementary.pdf]

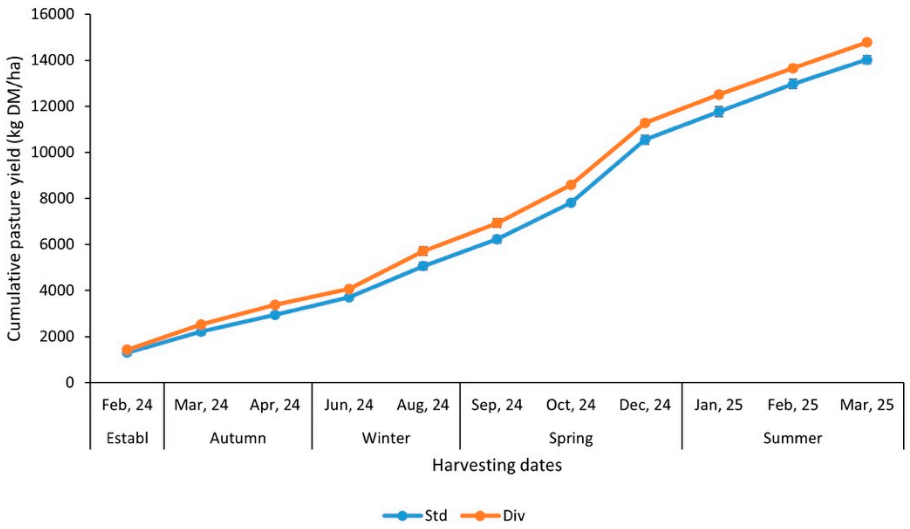

Figure S1: Cumulative dry matter production (kg DM ha^{-1}) of standard and diverse pasture treatments throughout the study. The error bar represents the standard error (n=6).

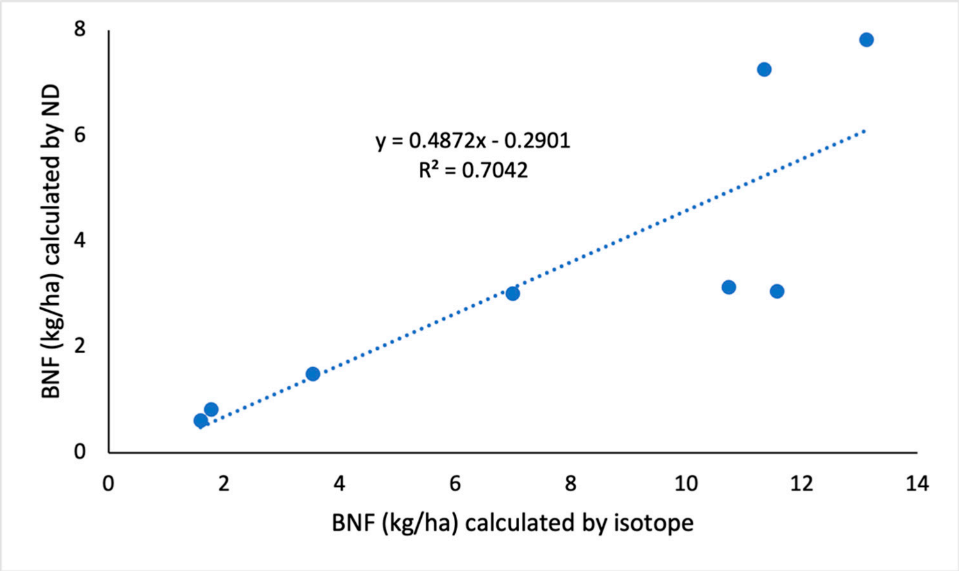

Figure S2: Correlation between biological nitrogen fixation (BNF) calculated using the isotope method and the nitrogen difference (ND) method. The scatter plot includes a fitted linear regression line ($y = 0.4872x – 0.2901$) with an R^2 value of 0.7042, indicating a moderate positive correlation between the two methods.
